# Organizational performance, Marketing strategy, and Financial strategic alignment: an empirical study on Iranian pharmaceutical firms

**DOI:** 10.1186/2008-2231-21-65

**Published:** 2013-08-02

**Authors:** Mehdi Mohammadzadeh, Sied Mohammad Aarabi, Jamshid Salamzadeh

**Affiliations:** 1 School of Pharmacy, Shahid Beheshti University of Medical Sciences, Tehran, Iran; 2 Sama Management Research Institute, Tehran, Iran

**Keywords:** Alignment, Marketing strategy, Financial strategy, Pharmaceutical, Performance

## Abstract

**Background:**

Strategic Functional-level planning should be aligned with business level and other functional strategies of a company. It is presumed that assimilating the strategies could have positive contribution to business performance, in this regard alignment between marketing strategy and financial strategy seems to be the most important strategies being studied. An empirical work in generic pharmaceutical manufacturing companies for evaluating effect of alignment between these two functions on organizational performance was developed in this paper.

**Methods:**

All Iranian pharmaceutical generic manufactures listed in Tehran stock market have been tested for period of five years between 2006–2010 and their marketing strategies were determined by using Slater and Olson taxonomy and their financial strategies have been developed by calculating total risk and total return of sample companies for five years based on rate of risk and return in the frame of a 2 × 2 matrix. For the business performance three profitability indices including Q-Tubin (Rate of market value to net asset value), ROA (Return on Asset), ROE (Return on Equity) have been tested. For analysis, a series of one-way ANOVAs as a collection of statistical models within marketing strategies considering financial strategy as independent variable and the three performance measures as dependent variables was used.

**Results:**

Results show strategic alignment between financial and marketing has significant impact on profitability of company resulting in arise of all three profitability indices. Q tubing’s rate were 2.33,2.09,2.29,2.58 and rate of ROA were 0.21,0.194,0.25,0.22 and rate of ROE were 0.44,0.46,0.45,0.42 for matched strategy types, respectively the rates shown here are more than average meaning that specific type of marketing strategy is fitted with specific type of financial strategy.

**Conclusion:**

Managers should not consider decisions regarding marketing strategy independently of their financial strategy.

## Introduction

Intense competition in an increasingly global environment has triggered renewed interest in the marketing function which its contribution can make competitive success to a company and this is possible if an appropriate matching exists between marketing and other functional activities of company.

In particular, it has been supposed that aligning financial and marketing strategies can make a company more productive and profitable. However, it has been discussed about aligning the functions of companies in recent years in both academic and trade publications [1-3], but little empirical researches have been conducted to determine effects of this alignment within industry defining how marketing strategies and financial strategies should support each other.

The results of an empirical work to determine the effect of alignment between marketing and financial strategies on organizational performance are discussed in this paper reporting that this empirical work exists within a sample of Iran’s pharmaceutical manufacturing companies.

### Strategic planning in theory

**Figure 1:**
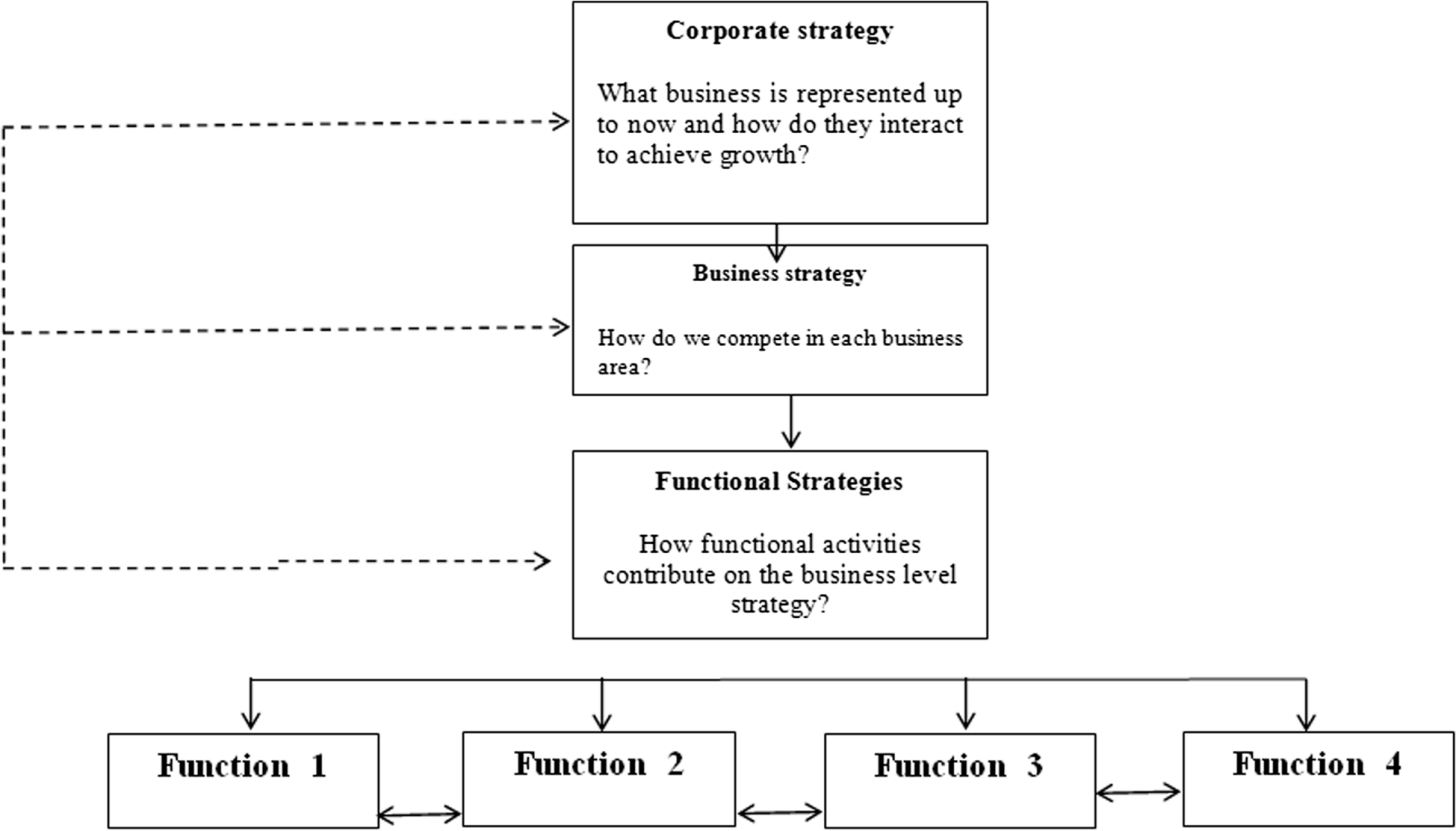
The strategy hierarchy.

Within an organization, strategies can be viewed at three levels as shown in Figure 1. These three levels of strategy from a hierarchy implies a top-down approach to strategy formulation. However, the three levels overlap and influence one another which they should be aligned with each other horizontally and vertically.

The development of strategies consisting of two distinct constituents can be thought: content and process. The content of strategy refers to the choices, plans and actions making up the strategy, but the strategy process are the act of formulating strategy, analysis, organization and implementation issues [4].

It has been focused on financial and marketing alignment and its contribution for organizational performance in pharmaceutical generic manufacturing companies.

### Marketing strategy

Marketing strategy is the set of integrated decisions and actions, [5] by which a company wants to recognize and meet customer needs in order to achieve marketing goals [6,7].

Marketing strategy is concerned with activities relating to context analysis at Macro and Micro level, competitor and customer analysis, segmentation, targeting, and defining an appropriate positioning based on marketing mix: product, price, place and promotion decisions [8-10].

The Concept of strategic marketing planning is based on the assumption that an organization should have a long-term outlook that is not only environmentally sensitive but also nationally and globally oriented. Therefore, when an organization develops its strategic policy it must go beyond its short or mid-term goals and must integrate feedback from stakeholders and customers to reach its goals and objectives bringing about benefit not only for the organization but also for consumers and society.

Hual and Madu (1994) presented four types of marketing strategies to reach a totality of strategic marketing orientation: Volunteer marketing, Total marketing, Value marketing and Environmentally conscious marketing [11].

Kotler (1991) presented a model of three types of marketing strategic orientation:External marketing, Internal marketing and Interactive marketing and explained how they are applied in today’s market place [12].

Hooley, Lyneh & Jobber [13] developed 5 types of marketing strategies for generic markets by extend the work by Porter [14] and Miles and Snow [15] by focusing on a wide range of marketing variables. They called as: Aggressors, Premium positioning segmentors, Stuck-in-the middlers, High value segmentors and Defenders.

Slater & Olson (2001) presented four types of marketing strategies with a comprehensive study of clustering marketing activities. They called their presented types of marketing strategies as: Aggressive Marketers, Mass Marketers, Marketing Minimizers and Value Marketers [16].

They defined this taxonomy according to difference of each type in following activities: market research, segmentation, product line breath, product innovation, product quality, customer service, premium pricing, selective distribution, advertising, internal sales force and support to promotion process [16].

In this study taxonomy of Slater and Olson has been represented for main purpose of defining types of marketing strategies in pharmaceutical sample companies.

### Financial strategies

West (2003) presented four type of financial strategies based on cost and revenue. He examined four financial strategies: (1) Uniform cost cuts (2) Selective cost reductions (3) Increased operational efficiencies and (4) Cost avoidance by shifting responsibilities to external parties [17].

Financial strategies are such a suitable frameworks of decision making dedicated to managers for increasing expected return and reducing cost and risk to meet organizational objectives.

As risk and return are two main factors in financial decision making, in this study four types of financial strategies based on required return and perceived risk, were perused. According to risk-return line, based on accepted risk, the required return will be changed expecting that with increase of accepted risk the return of company should be increased [18].

We named four types of financial strategies as:

1) Risk Accepting Strategy (RAc).

2) Conservative Strategy inclined to Risk Avoidance (CRAv).

3) Risk Avoidance Strategy (RAv).

4) Conservative Strategy inclined to Risk Accepting (CRAv).

According to the rate of risk and return, different financial strategies should be selected for a company, consequently decisions about main financial activities including Investment, Providing financial resource, cash flow and dividing of profit will be different as well.

### Organizational performance

In order to evaluate organizational performance, in this study three indices of Q-tubin, ROA and ROE were used that according to formulas shown in below and make use of extracted data from financial statements of companies these indices were calculated [19].

$$\text{Qtubin}=\frac{\left(\text{Equity}\;\text{Market}\;\text{Value}+\text{Liabilities}\;\text{Market}\;\text{Value}\right)}{\left(\text{Equity}\;\text{Book}\;\text{Value}+\text{Liabilities}\;\text{Book}\;\text{Value}\right)}$$

$$\mathrm{ROA}=\frac{\left(\mathrm{Net}\;\text{Income}+\text{Interest}\;\text{expense}\right)}{\text{Average}\;\text{assets}\;\text{during}\;\mathrm{the}\;\text{period}}$$

$$\mathrm{ROE}=\frac{\mathrm{Net}\;\text{Income}}{\text{shareholder}\;\text{equity}}$$

**Figure 2:**
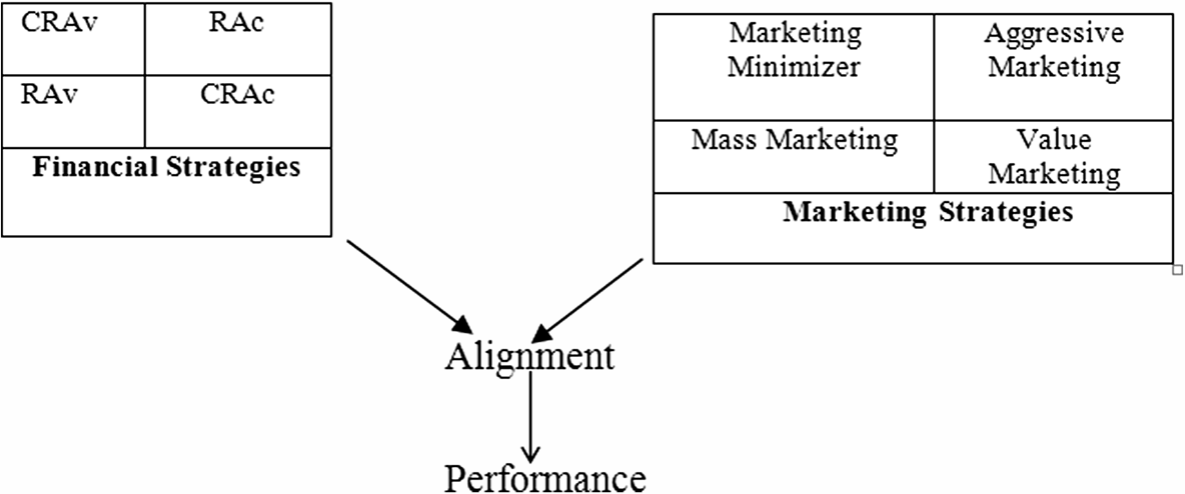
Conceptual model of alignment between marketing and financial strategies.

In this study strategic management theories are brought into the field of marketing and finance and put forward a model as shown in Figure 2, which explores the relationship between strategic alignment in field of marketing and financing with organizational performance. According to this model the following hypothesis were proposed:

### Alignment between marketing strategies and financial strategies is positively related to organizational performance and promote profitability

#### The study

As explained earlier, in this study Slater and Olson taxonomy for defining of marketing strategies type of sample companies was used. , they presented four types of marketing strategies as follow:

Aggressive Marketers, which explain firms that target the segment of buyers who prefer high quality, innovative products and who are willing to pay premium prices. They reach this selected group of buyers with a very selective distribution strategy, and invest in advertising and marketing support activities.

Mass Marketers, that offer a broad line of largely undifferentiated product. They utilize an intensive distribution strategy and charge low price.

Marketing Minimizers, which put comparatively little effort into marketing activity and provide lowest level of services for customer. In this type of strategy there are limited line of product, small amount of investment in marketing and innovation and prices are comparatively low, so it indicates that marketing is not a key factor in their value chains [14,15,20].

Value Marketers, in this type of strategy firms utilize selective distribution channel to provide high quality and innovative products with significantly lower prices in comparison to aggressive marketers. They usually use their own sales forces instead of advertising to communicate their value proposition.

### Measurements

The sample of this study indicates all Iranian pharmaceutical companies listed in Tehran stock market including 30 generic pharmaceutical manufacturing companies that provide more than 60 percent of Iran’s pharmaceutical market needs. In this regard, a period of five years between 2006–2010 were selected, which means 150 years assessing for defining of types of their strategies. Based on Slater and Olson study and considering nature of pharmaceutical marketing a questionnaire for determining strategies of marketing was developed as it is stated in Appendix, for adjusting questionnaire with pharmaceutical marketing an interview with 40 marketing managers or marketing experts who were involved in generic medicines marketing was done.

Financial strategies were defined by measuring of total risk and total return based on date reported in annually financial statements of companies and then performance indices calculated according to related formulas during the years study was being done, data was collected from financial reports as well.

### Marketing strategies

Marketing strategies were assessed using the Slater and Olson taxonomy by using the prepared questionnaire. To fill out the questionnaire, five marketing managers who had more than five years work experiences in the sample companies were selected from each company and the questionnaires were completed with face to face interview or on the phone, then for bias reduction the results of some respondents were submitted to another person to cross checking.

### Financial strategies

**Figure 3:**
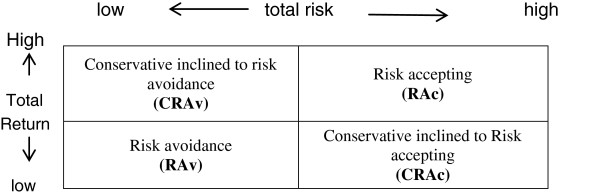
Diagram of financial strategy types based on risk and return.

For assessing financial strategies, total risk and total return as two strategic reference points for sample companies during the five years were determined, then average rate of risk and return of whole Iran’s pharmaceutical industry as the middle point were assigned and then the type of strategies was found based on the rate of total risk and total return (Figure 3).

As shown in Figure 3, if the rate of total risk and total return be more than the average rate of these indices in industry, the type of strategy would be Risk Accepting (RAc), if the total risk be more than average rate and total return be lower than it, then the strategy would be Conservative inclined to Risk Accepting (CRAc), also if total risk be lower than average rate and total return be more than it, the strategy type would be Conservative inclined to Risk Avoidance (CRAv); finally if both total risk and total return be lower than average rate, the strategy would be Risk Avoidance (RAv).

### Performance

Business performance is a complex and multi–faceted concept. In this study, three financial indicators including Q-tubin, ROA, ROE indices were used to assess profitability and market added value of sample Companies.

The average of industry was defined as middle point and the rate above average as good performance and the rate below average as weak performance were measured, respectively.

### Validity and reliability

For the validity testing of questionnaire, content validity with assessing of comprehensiveness, relevance and clarity of each question based on pharmaceutical marketing experts’ opinion was evaluated.

For the reliability evaluation, Cronbakh’s alpha coefficient for each question was calculated, and then by modifying the text and form of items, rate of coefficient to minimum 0.77 was achieved.

### Analysis

To assess the benefit of alignment between financial strategy and marketing strategy types, a series of one-way ANOVA software within marketing strategies were used, for that, financial strategy as independent variable and the three performance measures as dependent variables, were used. The least significant difference test to detect significant differences (at p < 0.05) between financial strategy types within each marketing strategy type was applied. T-tests to perform pairwise companies between all groups means were applied for those least significant differences were used as well.

## Findings

The results of this analysis, as shown in Table 1 are quite compelling. Indeed, these results indicate strong support the effect of matching strategies on profitability indices.

**Table 1:** Performance implications of the match between marketing strategy and financial strategy

**Q-tubin**	RA	CRAv	RA	CRAc
Aggressive marketing	**2.33 (0.54)**	1.84 (0.38)	1.63 (0.36)	1.76 (0.34)
Marketing minimizer	1.64 (0.36)	**2.09 (0.28)**	1.74 (0.35)	1.43 (0.29)
Mas marketing	1.66 (0.42)	1.72 (0.34)	**2.29 (0.41)**	1.82 (0.36)
Value marketing	2.11 (0.42)	2.09 (0.46)	2.18 (0.44)	**2.58 (0.62)**
**ROA**	RA	CRAv	RA	CRAc
Aggressive marketing	**0.21 (0.07)**	0.10 (0.03)	0.12 (0.03)	0.15 (0.04)
Marketing minimizer	0.08 (0.02)	**0.194 (0.05)**	0.11 (0.03)	0.09 (0.04)
Mas marketing	0.11 (0.04)	0.07 (0.02)	**0.25 (0.08)**	0.13 (0.03)
Value marketing	0.10 (0.02)	0.14 (0.03)	0.15 (0.04)	**0.22 (0.05)**
**ROE**	RA	CRAv	RA	CRAc
Aggressive marketing	**0.44 (0.10)**	0.34 (0.08)	0.37 (0.08)	0.35 (0.07)
Marketing minimizer	0.36 (0.11)	**0.46 (0.09)**	0.38 (0.08)	0.34 (0.07)
Mas marketing	0.39 (0.08)	0.32 (0.06)	**0.45 (0.11)**	0.35 (0.08)
Value marketing	0.32 (0.06)	0.34 (0.07)	0.37 (0.09)	**0.42 (0.08)**

Means performance value/S.D.

Financial strategy type differences into marketing strategy type. The bold numbers show the higher performance of matched strategy types.

As shown in Table 1 all three performance indices are significantly higher in matched strategy types, it means special pairwise strategy types indicate higher range of profitability.

## Discussion

Strategies in all levels within the organization should be compiled and support each other regarding the thorough goals existing in the organization to creating synergy. In spite of the fact that design and compile of strategy within an organization is essential with top-down or bottom-up approach, objectives, operations and the method for implementing. The top level strategies should be reflected in bottom levels and for this purpose the strategies of all levels must be matched or aligned with together. Through this, functional levels have very essential role in developing strategic coordination. Along this, each of the functional sectors have to had relevancy with each other even horizontally and vertically and this is in a way that activity of them causes reinforcement of the others resulting improvement of the organization’s performance. Marketing as brain of the company exists to find needs and wants of customer to meet them and achieve satisfaction of customer to maintain and develop market. On the other hand financing sector exist to organize providing of essential resources for organization, developing the investment provision and development and restraining the excess costs and exploitation of resources in the organization. Marketing unit has to investigate the current situation of the financial unit before acting for designing the plans. In this study, marketing and financial strategies have been divided into four different types and the specific definition of them for the purpose of alignment assessment has been represented; in this case, the obtained results show that each of the marketing strategies is relevant with financial strategy and specific pairwise strategies cause more performance for organization, so based on results the four below propositions can be reported.

Proposition 1. Aggressive Marketer will achieve superior performance in case of utilizing a Risk Accepting financial strategy.

Proposition 2. Mass Marketers will achieve superior performance in case of utilizing a Risk Avoidance financial strategy. Results have shown that most of the pharmaceutical companies which are active in field of generic products use mass marketing and risk avoidance strategies for marketing and financing. Perhaps having similar forms and name for products across the market trigger decisions of this type.

Proposition 3. Marketing Minimizer will achieve more performance in case of utilizing a Conservative inclined to Risk Avoidance financial strategy.

Proposition 4. Value Marketers will achieve superior performance in case of utilizing a Conservative inclined to Risk Accepting financial strategy.

While the results from this study support the proposition that generic pharmaceutical manufactures that have internally consistent plans for marketing and financial strategies are more profitable than those without consistency, there may be other factors influencing this relationship. Perhaps future research could investigate the relationship between other functional strategies like human resources, information system and operation with marketing and financial strategies.

## Conclusion

Generic pharmaceutical product marketing is one of the domains within pharmaceutical marketing with high competitiveness in designing, compiling and implementing the strategy relevant with objectives and the situation of the market. Managers in the field of both generic and ethical pharmaceutical marketing have to be informed of the fact that decision making and determining the strategy in any level of the organization have to be aligned with higher and/or lower levels. As the results of this study illustrates the role of financial strategy in the marketing strategy decision and it means that managers should not consider decisions regarding marketing strategy independently of their financial strategy.

## Appendix

***Question****: According to the years mentioned below, which of the following options offers the best description of the marketing strategy of your company? Please check the box beside each option (year) which matches to the description most.*

Each option can be determined for one year or more yet for each year one option (strategy) can be selected. In other words, strategy can be the same for different years but for one year you cannot select 2 strategies.

1. *Our company proceeds with creative activities and presenting new products of high quality, having a close relationship with customers including prescribers, patients, influencers,(KOL) key opinion leaders, and retailors. The company has carried out a widespread marketing research in order to find opportunities, threats, as well as analyzing customers and competitors and try to present the products with prestigious prices. Our distributing strategy is selective, therewith those customers and/or markets which are able to afford at high stand are targeted. Professional and efficient marketing staff are employed. We vary our products frequently and support a large number of scientific and medical incidents and conferences for physicians, and pharmacists. 2006? 2007? 2008? 2009? 2010?*

2. *Our company presents the products of appropriate quality and performs as follower for innovation and product development. A widespread channel of distribution is applied for distributing of products. We compete by using of lower price and financial encouragement instead of advertising and professional marketing. We put emphasis on promotion of the sale of our present products more than its diversity and differentiation. Besides, a number of the marketing staff work to establish a relationship with customers. 2006? 2007? 2008? 2009? 2010?*

3. *Preceding the presentation of the products to the market, our company waits for it to see if the concept of product is accepted by the customers, in this way we decrease the risk. We manage our market through the accepted quality, lower price and strategy of widespread distribution. We highly focus on the production lines and pursuit the productive and low cost production. We mostly take the benefit of sales staff rather than the marketing ones. 2006? 2007? 2008? 2009? 2010?*

4. *Our company presents the products of high quality and innovative. Establishing a close relationship with the customers including influencers, Key Opinion Leaders, retailors (pharmacies) to value creation. The company performs less systematic marketing strategy and hardly turns to products with high value, prestigious pricing, or high affordable markets. Distributing strategy is not totally selective or global yet something in between. Advertising and marketing is taken into account moderately. 2006? 2007? 2008? 2009? 2010?*

## Competing interests

The authors declare that they have no competing interests.

## Authors’ contributions

SMA carried out designing and conceptual modeling of study. JS helped to draft the manuscript, participated in questionnaire forming. MM participated in design of the study and performed the statistical analysis. All authors read and approved the final manuscript.
